# Gender Differences in State Anxiety Related to Daily Function Among Older Adults During the COVID-19 Pandemic: Questionnaire Study

**DOI:** 10.2196/25876

**Published:** 2021-06-03

**Authors:** Sara Rosenblum, Ortal Cohen Elimelech

**Affiliations:** 1 The Laboratory of Complex Human Activity and Participation Department of Occupational Therapy University of Haifa Haifa Israel

**Keywords:** COVID-19, coronavirus, anxiety, cognition, aging, eHealth, online data

## Abstract

**Background:**

The COVID-19 pandemic poses a challenge to people’s day-to-day functioning and emotional and physical health, especially among older adults.

**Objective:**

The aim of this study is to analyze gender differences in state anxiety, daily functional self-actualization, and functional cognition as well as the relationships among those factors in older adults during the COVID-19 pandemic lockdown.

**Methods:**

We collected data on the web from a sample of 204 people (102 men and 102 women) aged 60 years and older. In addition to a demographic questionnaire, we used the State-Trait Personality Inventory to assess state anxiety, the Daily Functional Actualization questionnaire to evaluate daily functional self-actualization, and the Daily Living Questionnaire to measure functional cognition.

**Results:**

Significant gender differences were found for state anxiety (*t*_202_=−2.36*, P*=.02); daily functional self-actualization (*t*_202_=2.15, *P*=.03); and the functional cognition components: complex tasks (*Z*=−3.07, *P*=.002); cognitive symptoms that might be interfering (*Z*=−2.15*, P*=.028); executive functions (*Z*=−2.21, *P*=.024); and executive function monitoring (*Z*=−2.21, *P*=.027). Significant medium correlations were found between both state anxiety level and daily functional self-actualization (*r*=−0.62, *P*<.001) and functional cognition (*r*=0.37-0.40*, P*<.001). Gender predicted 3% of the variance in state anxiety level, while daily functional self-actualization predicted 41% and complex activities (Daily Living Questionnaire) predicted an additional 3% (*F*_3,200_=58.01, *P*<.001).

**Conclusions:**

In older adults, anxiety is associated with cognitive decline, which may harm daily functional abilities and lead to social isolation, loneliness, and decreased well-being. Self-awareness and knowledge of gender differences and relationships between common available resources of daily functional self-actualization and functional cognition with anxiety may be strengthening factors in crisis periods such as the COVID-19 pandemic.

## Introduction

### Background

The COVID-19 pandemic poses a challenge to people's day-to-day functioning and constitutes a meaningful factor related to their emotional and physical health [1,2]. Expansion of the disease, reports of illness and death, and social isolation, especially during lockdown, may lead people to feel sadness, depression, and anxiety and to decrease their daily functioning [3,4]. Among older adults, this situation is especially worrisome. Anxiety disorders and symptoms have been found to be the most widespread mental disorders among this population, even when not in crisis periods. Further, such disorders and symptoms may worsen physical resistance and disease in older adults [5,6].

Within the literature, some studies have focused specifically on mental health of older adults during the COVID-19 pandemic crisis. These studies evidenced increased depression symptoms and anxiety stress among older persons, with higher levels of distress found among women [7-9]. For example, approximately 12% of 7127 older adults in a survey conducted in the United Kingdom from May to July 2020 reported feeling worse depression and anxiety levels during the COVID-19 lockdown; of those reporting worse depression and anxiety, 17% were women, while only 7.8% were men [7]. Gustavsson and Beckman [9] reported significantly higher frequency of decreased mental health among older women than among men of the same ages, such as feeling bad due to isolation, experiencing worries (about health, the economic situation, and implications to society), having sleeping problems, and feeling depressed. Casagrande et al [8] and Robb et al [7] summarized that older women had increased risk for higher levels of distress and anxiety compared to older men.

Although these findings described the mental health status of older adults during the COVID-19 pandemic, there remains a general lack of literature about gender-related relationships between mental health, specifically anxiety levels and daily function characteristics. In routine periods, interrelationships were described between satisfying daily function and cognitive functioning with aging well and lower anxiety levels among older persons [10-12]. Older people who were active, satisfied with their daily function, and had no cognitive limitations interrupting their daily function achieved better mental health, including the lowest anxiety [11]. On the other hand, studies found associations among older adults between declined or unsatisfactory functional abilities loss of control and decreased physical and mental health [13]. Because gender differences were reported as prominent in mood and anxiety disorders [14], this factor must be considered when analyzing daily function as it relates to anxiety.

Crisis periods especially increase the importance of discovering mechanisms that enable people to maintain or achieve good, durable mental health. Thus, this study focuses on analyzing state anxiety as it relates to two further concepts that reflect older adults’ daily functioning: (1) daily functional self-actualization and (2) functional cognition. *State anxiety* is defined as a temporary emotion characterized by feelings of tension and increased autonomic nervous system functions in situations experienced as a threat and that continues as long as the situation is interpreted to be a threat [15]. Teixeira et al [16] reported higher levels of state anxiety among women ages 62-93 in routine times (eg, not during crises such as the COVID-19 pandemic) than among men of the same ages.

Maslow [17] described *self-actualization* in his 1954 hierarchical motivation theory as individuals’ expression of their full potential and desire for self-fulfillment. His growth-focused actualization model emphasized individual strengths towards a growth-oriented approach rather than a pathology-oriented approach focused on individuals’ deficiencies [18]. In this study, we consolidated the Maslow self-actualization theory [17] with concepts of daily functional self-actualization based on the World Health Organization (WHO) International Classification of Functioning, Disability, and Health (ICF) concepts [19]. The WHO established the ICF as a model of health and disease management linking health-related issues with individuals’ capacities, daily activity performance, and participation and environmental factors. Health-related issues include physical and psychological conditions that affect a person's daily functional abilities and vice versa. Thus, satisfying daily functioning can lead to improved sense of control, homeostasis, and better health [20]. Combining Maslow's self-actualization theory with the ICF classification concepts and further literature (eg, [21,22]) enabled us to assume that if individuals are satisfied with their daily doing (activities and participation as related to their environmental expectations [19]) and feelings of mastery and competence while achieving their full potential [17], then they may feel less anxiety due to loss of life control and more self-fulfilled despite the COVID-19 crisis. Thus, self-actualization related to daily doing—that is, *daily functional self-actualization*—may be a source of strength for older adults during crises. However, as far as we know, no previous study has reported gender differences related to this concept, likely due to the absence of a practical standardized instrument to measure them. Consequently, although previous results indicated significant relationships between either daily functional abilities or individuals’ self-fulfillment and health and well-being (eg, [21,22]), relationships between daily functional self-actualization and state anxiety among older people have not been studied, or at least have not been reported.

A further aspect analyzed in the current study in the context of searching for strengthening factors is functional cognition. *Functional cognition* refers to the application of cognitive skills to self-care and community living. Life consists of a process of daily cognitive problem-solving requirements that are essential to efficiently accomplish daily activities. Efficient performance can engender feelings of daily occupational satisfaction [23]. The cognitive ability to perform routine tasks and complex everyday activities entails functional cognition [24]; thus, functional cognition is properly evaluated in the context of actual task performance. It includes individuals’ capacity to perform tasks and considers their abilities as a whole, including habits, routines, and environmental resources [24].

Beyond the differences related to anxiety that prior studies have reported, our review of the literature revealed that gender differences in daily functional self-actualization or in functional cognition have not been described among older adults during crisis periods.

### Research Aims

Considering the possible implications of daily functional self-actualization on anxiety and health, recognizing gender differences and understanding relationships among these concepts may supply insight into each gender’s unique needs. This may then provide opportunities to improve both older adults’ well-being in crisis periods and stakeholders’ awareness. As such, this study aimed to analyze gender differences related to state anxiety, daily functional self-actualization, and functional cognition, as well as the relationships among those factors, in older adults during the COVID-19 pandemic lockdown. We hypothesized that (1) significant gender differences would be found in state anxiety, daily functional self-actualization, and functional cognition; (2) significant correlations would be found among state anxiety, daily functional self-actualization, and functional cognition for the entire sample; and (3) gender, daily functional self-actualization, and functional cognition would predict state anxiety for the entire sample.

## Methods

### Participants and Procedure

The data were collected from 204 people (102 men and 102 women) aged 60 years and older between September 20 and October 10, 2020—a period when the respondents were in lockdown due to the COVID-19 pandemic. The data were gathered using an Israeli survey company, Panel4, that specializes in recruiting and incentivizing participants for web-based research (eg, in exchange for gift certificates). Panel4 can provide 400,000 panel members recruited via advertisements on Google, Facebook, and other popular websites, thus representing the adult population in Israel for web-based research. Ours was a sample of those panelists who chose to participate in response to our call distributed by the company among their members. Participants completed a demographic questionnaire and the instruments detailed in the following sections anonymously and independently on the web (via a link to the Qualtrics platform).

### Instruments

#### State Anxiety: State-Trait Personality Inventory

State anxiety was measured by the State-Trait Personality Inventory (STPI) [25], a 10-item inventory that assesses the respondent’s specific pattern of emotions, such as anxiety, anger, depression, and curiosity. Participants rate their present agreement with the items on a 5-point Likert-type scale from 1 (not at all) to 5 (very much). Higher scores indicate greater state anxiety (CD Spielberger, G Jacobs, R Crane, et al, 1979, *Preliminary manual for the State-Trait Personality Inventory (STPI)*, University of South Florida, unpublished manuscript). In this study, the STPI internal consistency was high (.71), which is consistent with previous studies [5,25]. Moreover, the STPI has been found to have good reliability and validity among older adults [26].

#### Daily Functional Self-Actualization Questionnaire

**Figure 1:**
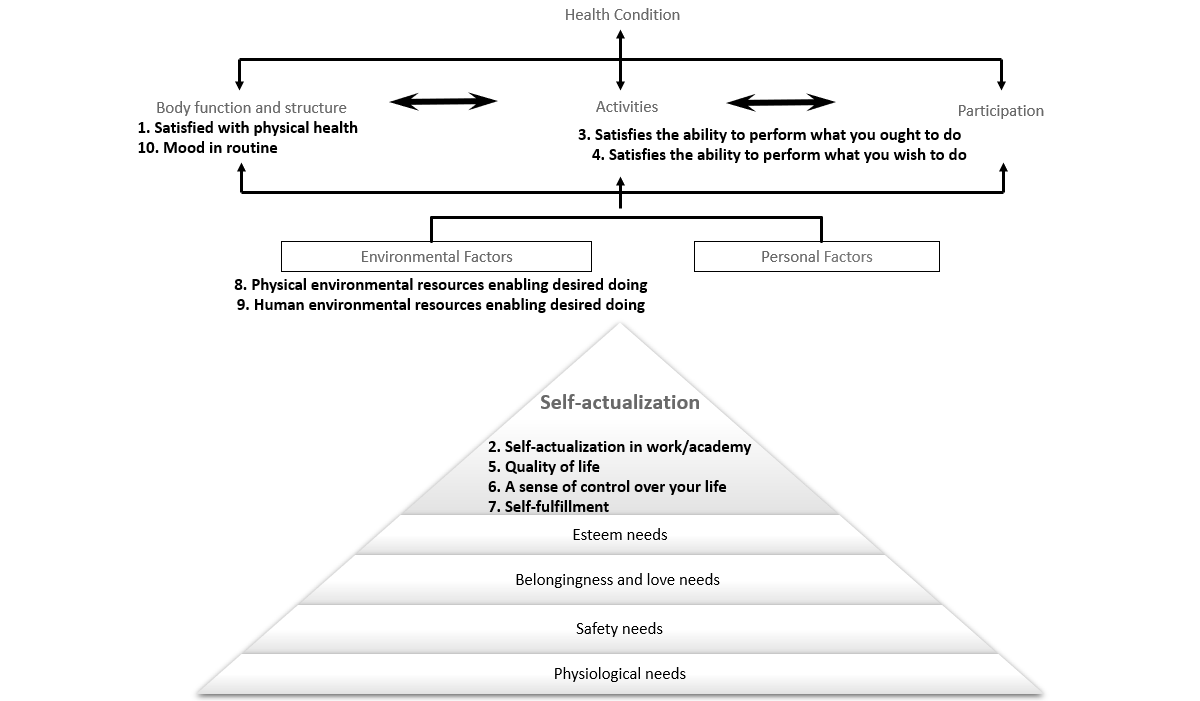
The 10 items of the Daily Functional Actualization questionnaire, derived from both the International Classification of Functioning, Disability, and Health [19] and Maslow’s theoretical concepts [17].

The Daily Functional Self-Actualization Questionnaire (DailyFA) was developed by the authors within a short time in response to the need to measure daily self-actualization during the COVID-19 lockdown period. Based on the Maslow theory [17], the ICF concepts [19], and the first author’s rich clinical experience, results of studies among the aging population (eg, [20,27-29]) and development of 11 assessment tools, the first author chose 10 items for the questionnaire. These items as derived from both the ICF [19] and Maslow theory [17] (see Figure 1) related to physical and emotional health, sense of control over life, satisfaction from daily doing, self-fulfillment, environmental factors, and quality of life. Content and face validity were established based on literature reports about self-actualization and daily function. Expert validity was further established by four occupational therapy researchers, who agreed that the items are appropriate to cover the construct of daily functional self-actualization and that each item was clearly articulated. Participants rated their fulfillment or satisfaction with each item on a 10-point scale from 1 (not good at all) to 10 (very good). The DailyFA indicated a high level of internal consistency (α=.94) in this study.

#### Functional Cognition: Daily Living Questionnaire

In this study, we measured functional cognition using the self-report Daily Living Questionnaire (DLQ), which relates to cognitive difficulties in activities and participation as well as to cognitive components such as memory, executive functions, and executive function monitoring [27]. The DLQ assesses everyday difficulties in activities and participation tied with higher level cognitive deficiency. It includes 51 daily activities within 2 domains. The first domain, Part A (28 items), relates to activities and participation and consists of 4 factors: household tasks, activities involving language/comprehension, community/participation, and complex tasks. The second domain, Part B (24 items), relates to cognitive symptoms or impairments and consists of 3 factors: executive function, memory, and executive function monitoring. Using a 4-point Likert-type scale from 1 (no mental or cognitive difficulty) to 4 (unable to complete), respondents rate their level of mental or cognitive difficulty when performing activities. The DLQ content and face validity were established, and it was found to have an acceptable internal consistency for both parts. Furthermore, validity has been demonstrated by distinctions between age groups and between participants with multiple sclerosis and controls [20]*.* The internal consistency for this study was high for all 52 items (α=.94) and for each part separately (α=.87 for Part A; α=.94 for Part B).

### Data Analysis

Descriptive statistics were used to describe the sample. The demographic gender variables (*woman* and *man*) were compared using chi-square tests, *t* tests for independent variables, and Mann-Whitney tests. Between-group differences in state anxiety, daily functional self-actualization, and functional cognition were examined using *t* tests for independent variables and Mann-Whitney tests. To examine the prediction of state anxiety by gender, daily functional self-actualization (DailyFA mean score), and functional cognition (DLQ factors), we conducted stepwise regressions, with gender inserted at the first phase and DailyFA and DLQ variables afterward. 

## Results

### Sociodemographic Characteristics

The sample included 204 Jewish adults living in Israel. Most spoke Hebrew (94/102 women, 92.2%; 96/102 men, 94.1%) and lived in a city (94/102 women, 92%; 84/102 men, 82%). As presented in Table 1, the participants’ sociodemographic characteristics indicated no significant gender differences for age, years of education, place of birth, main language spoken, place of residence, or religious affinity. However, significantly fewer female than male participants were married (with or without children) or were retired. No significant correlations were found between marital status or being retirement and any of the main research variable scores (eg, state anxiety, daily functional self-actualization, or functional cognition factors). Thus, those sociodemographic characteristics were not retained as covariates in the later gender-comparison analyses.

**Table 1:** Participants’ sociodemographic characteristics by gender (N=204).

Sociodemographic characteristic		Women (n=102)	Men (n=102)	χ^2^ (*df*)		*P* value	
Age (years), mean (SD)		69.01 (0.58)	69.28 (0.59)	0.33^a^ (202)		.74	
Education (years), mean (SD)		13.6 (0.44)	13.69 (0.42)	0.09 (201.69)		.93	
Years since retirement, mean (SD)		11.28 (7.57)	10.64 (9.15)	−0.98^b^		.32	
**Place of birth, n (%)**					9.05 (6)		.17
	Israel	72 (70.6)	63 (61.8)				
	Europe	8 (7.8)	19 (18.4)				
	United States	1 (1.0)	1 (1.0)				
	Asia	6 (5.8)	1 (1.0)				
	Africa	5 (4.9)	5 (4.9)				
	Former Soviet Union	6 (5.8)	8 (7.8)				
	Other	4 (3.9)	5 (4.9)				
**Marital status, n (%)**					25.27 (4)		<.001
	Single	5 (4.9)	5 (4.9)				
	Separated or divorced	28 (27.5)	8 (7.8)				
	Married with or without children	59 (42.2)	88 (69.2)				
	Widowed	10 (9.8)	1 (1.0)				
**Religious affinity, n (%)**					5.65 (3)		.13
	Secular	75 (73.5)	71 (69.9)				
	Religious	2 (2.0)	9 (8.8)				
	Ultra-orthodox	1 (1.0)	0 (0)				
	Traditional	24 (23.5)	22 (21.6)				
**Employment status, n (%)**					2.82 (1)		.09
	Usually working	46 (45.1)	58 (56.9)				
	Not usually working	56 (54.9)	44 (43.1)				
**Retirement, n (%)**					6.72 (1)		.01
	Yes	30 (29.4)	48 (47.1)				
	No	72 (70.6)	54 (52.9)				

^a^*t* test.

^b^Mann-Whitney test.

### Gender Differences: Hypothesis 1

#### State Anxiety

Independent *t* tests showed significant group differences for state anxiety based on STPI score. The female participants had significantly higher mean state anxiety scores than the male participants (female: mean 24.98, SD 7.98; male: mean 22.44, SD 7.31; *t*_202_=−2.36, *P*=.02).

#### Daily Functional Self-Actualization

Independent *t* tests revealed that during the COVID-19 crisis period, the female participants had significantly lower mean DailyFA scores than the male participants (female: mean 6.06, SD 1.96; male: mean 6.64, SD 1.94; *t*_202_=2.15, *P*=.03), indicating lower daily functional self-actualization for the women. The means and standard deviations for each DailyFA item are presented in Table 2.

**Table 2:** Gender comparison of means and standard deviations of the DailyFA questionnaire items and final mean scores.

DailyFA^a^ item	Score, mean (SD)	
	Women (n=102)	Men (n=102)
1. Satisfied with physical health	7.37 (2.13)	7.06 (1.94)
2. Self-actualization in work/academy	6.01 (2.85)	6.70 (2.63)
3. Satisfies the ability to perform what ought to do	6.58 (2.90)	6.83 (2.36)
4. Satisfies the ability to perform what you wish to do	5.51 (2.55)	5.71 (2.51)
5. Quality of life	6.01 (2.19)	6.74 (2.09)
6. A sense of control over your life	5.82 (2.42)	6.47 (2.51)
7. Self-fulfillment	5.10 (2.53)	5.85 (2.65)
8. Physical environmental resources enabling desired doing	5.67 (2.52)	6.27 (2.51)
9. Human environmental resources enabling desired doing	5.95 (2.61)	6.58 (2.42)
10. Mood in routine	6.24 (2.50)	6.97 (2.07)
Total mean score	6.03 (1.97)	6.65 (1.94)

^a^Daily FA: Daily Functional Actualization questionnaire.

#### Functional Cognition

As presented in Table 3, the Mann-Whitney analysis showed that women reported significantly more cognitive difficulties while performing both community-related and complex tasks. They also reported higher cognitive symptoms that might be interfering with daily performance on the whole DLQ, on the Part B score, and on each of its components (executive function, memory, and executive function monitoring).

**Table 3:** Gender comparison of the mean scores and standard deviations of the DLQ factors.

DLQ^a^ factor		Score, mean (SD)			*Z*		*P* value
		Women (n=102)	Men (n=102)				
**Part A: Activities and participation**		1.56 (0.46)	1.54 (0.52)	−8.13		.43	
	1. Household task	1.29 (0.38)	1.46 (0.57)	−1.66		.11	
	2. Activities involving language/comprehension	1.54 (0.54)	1.52 (0.62)	−0.76		.45	
	3. Community/participation	1.75 (0.71)	1.69 (0.61)	−1.01		.35	
	4. Complex task	1.74 (0.71)	1.49 (0.61)	−3.07		.002	
**Part B: Cognitive symptoms that might be interfering**		1.54 (0.52)	1.41 (0.49)	−2.15		.03	
	1. Executive function	1.62 (0.58)	1.47 (0.56)	−2.21		.02	
	2. Memory	1.43 (0.55)	1.44 (0.64)	−1.65		.08	
	3. Executive function monitoring	1.46 (0.52)	1.33 (0.45)	−2.21		.03	

^a^DLQ: Daily Living Questionnaire.

### Correlations: Hypothesis 2

Spearman correlations yielded significant correlations between state anxiety and both the daily functional self-actualization score on the DailyFA (*r*=−0.62) and some functional cognition factors on the DLQ (complex task: *r*=0.37; executive function: *r*=0.37; and executive function monitoring: *r*=0.40), with *P* values of <.001 for all 4 variables.

### Predictors of State Anxiety: Hypothesis 3

Tables 4 and 5 present the stepwise regression analysis, which indicates that gender contributed 3% to anxiety prediction. Daily functional self-actualization accounted for 41% of anxiety prediction, and the functional cognition subscale, *complex activities*, accounted for an additional 3%. Thus, 47% of the state anxiety variability was predicted by gender, daily functional self-actualization, and complex activities (*F*_3,200_=58.01, *P*<.001).

**Table 4:** Predicting state anxiety from gender, daily functional self-actualization, and functional cognition.

Value	Model 1				Model 2				Model 3		
	B (SE)	β	*P* value	B (SE)		β	*P* value	B (SE)		β	*P* value
Gender^a^	2.59 (1.07)	.16	.02	1.03 (0.82)		.07	.21^b^	0.67 (0.82)		.04	.41
Daily functional actualization (DailyFA)^c^	N/A	N/A	N/A	–2.55 (0.21)		–.65	<.001	–2.33 (0.22)		–.60	<.001
Complex activities (DLQ)^d^	N/A	N/A	N/A	N/A		N/A	N/A	1.98 (0.64)		.17	.002

^a^Gender 1=female.

^b^N/A: not applicable.

^c^DFA: Daily Functional Actualization questionnaire.

^d^DLQ: Daily Living Questionnaire.

**Table 5:** F and *R* values of the statistical analysis predicting state anxiety from gender, daily functional self-actualization, and functional cognition.

Value	Model 1	Model 2	Model 3
F change	5.57^a^	148.09^b^	9.57^c^
*R*^2^ (adjusted *R*^2^)	0.03 (0.02)	0.44 (0.43)	0.47 (0.46)
*R*^2^ change	0.03	0.41	0.03

^a^*P*=.02

^b^*P*<.001.

^c^*P*=.002

## Discussion

### Principal Results

This study aimed to evaluate gender differences related to the state anxiety, daily functional self-actualization, and functional cognition of older adults during the COVID-19 lockdown period and to search for relationships among those factors. The results show significant gender differences in all three areas and significant correlations between state anxiety and both daily functional self-actualization and some functional cognition components. Further, 47% of the state anxiety variability was predicted by gender (3%) and both daily functional self-actualization (41%) and the complex activities subscale of the DLQ measuring functional cognition (3%).

### Gender Differences

Anxiety is associated with cognitive decline and may harm daily functional abilities, which in turn can lead to loneliness and decreased well-being [5,30]. The results show significantly higher state anxiety levels among women during the pandemic period. These findings are supported by previous studies conducted in the United Kingdom [7], Italy [8], and Sweden [9], which presented significantly more negative feelings—including anxiety—among women than among men. Similarly, the significant gender differences found in daily self-actualization, which represents people's resources and feelings related to their daily function, are supported by studies that focused on gender differences in functional capacity and abilities in routine periods and not during crises such as the COVID-19 pandemic. These studies found gender differences in actual daily performance. However, they did not measure daily functional self-actualization in routine periods or in the COVID-19 pandemic period. Thus, a comparison of these results with previous studies is limited. The earlier findings included a study by Potvin and colleagues [26], which reported that women over the age of 65 years had significantly lower scores on performance-based functional capacity tests.

Gender differences have also been found in activities of daily living and instrumental activities of daily living performance among people aged 82 to 87 years [31]. Pachana and colleagues [31] noted that despite a higher incidence of chronic conditions among women, the female participants in their study reported significantly less difficulty preparing meals, taking medications, using the telephone, and performing leisure activities than the male participants. However, the women reported having significantly greater difficulty with shopping and housework activities compared to the partnered men.

Some difficulties performing such activities may relate to decreased executive functions. Executive functions are a set of cognitive skills that are necessary to plan, monitor, and execute a sequence of goal-directed complex actions [32]. Decreases in those abilities need to be identified in a timely fashion because they may influence daily functional performance abilities [33]. For example, Coppin and his colleagues found an association between poorer executive function and lower gait speed among older adults [34]; this may serve an important marker for decreases that may be associated with both physical and mental health. Interestingly, when focusing on functional cognition, the results of this study indicate significantly more functional cognitive difficulties for women in “complex tasks” and in cognitive components, such as executive function and executive function monitoring. However, these results did not hold for other kinds of tasks, such as household tasks (as also reported by Pachana and colleagues [31]) or for activities involving language and community/participation. It is noteworthy that Pachana and colleagues [31] reported a higher incidence of chronic conditions among women. It would be interesting to study the possible associations between difficulty performing complex daily tasks—which are cognitively demanding and require massive decision-making and thus demand daily energy—and women’s physical and mental health status. Analysis of a more comprehensive model that considers possible moderators and mediators variables is required.

Indeed, difficulties performing daily tasks in older adults may be tied with emotional, physical, or cognitive deficits [28]. Specifically, our results show significant inferior cognitive symptoms that may be interfering in daily function (eg, in the executive function and executive function monitoring of the DLQ Part B) among women. Similar to the results of this study, earlier studies showed that women older than 65 years and living in the community exhibited lower cognitive performance on the Mini-Mental State Examination (MMSE) than men in the same circumstances [35,36]. In contrast to our results, McCarrey and colleagues [37]—who used the MMSE and other neuropsychological tests—reported decreasing executive function abilities with age and greater resilience to age-related cognitive decline in older women than in older men. In the same line, in their comprehensive review of studies of gender comparisons related to executive functions, Grissom and Reyes [38] presented no gender differences in executive function as measured by neuropsychological tests. The MMSE is designed to screen for cognitive decline, and it (or similar neuropsychological tests) may not reflect real-world functional cognition [39]. These opposing results related to older adults’ abilities in specific cognitive components suggest a reminder that our study focused on *functional* cognition. From an ecological viewpoint, life becomes more complex and demanding over the years. Among older adults, especially in late life, extensive impairment of daily functioning may occur due to cognitive decline [40]. Indeed, measuring cognitive difficulty levels when performing varied tasks adequately reflects such reduction in daily life functioning [20]. Consequently, further studies are required to determine whether the significantly inferior executive function and executive function monitoring found in our research among women is due to the use of a cognitive functional self-report scale or caused by the crisis period, which may have more influence on the women’s functional behavior.

### Relationships Among State Anxiety, Daily Functional Self-Actualization, and Functional Cognition

The question of relationships among functional cognition, daily functional self-actualization, and state anxiety—especially in crisis periods—is of interest toward better understanding and possible prevention. The significant correlations found in our study are supported by previous studies. For instance, earlier studies found that women with generalized anxiety disorder presented higher degrees of functional impairment [41,42]. Although better cognitive performance has been found to be a protective factor among older adults [43], anxiety can cause an increase in cognitive dysfunction [44]. Prior research also associated community-dwelling older adults who showed higher state anxiety with worse learning, memory, and executive function abilities [45,46]. A long-term longitudinal study among twins over the age of 50 years reported a bidirectional relationship between anxiety symptoms and certain cognitive abilities, including processing speed and attention [23]. Additional cognitive performance, such as visuospatial ability and immediate and delayed memory, have also been found to be significantly associated with both state and trait anxiety among healthy older adults [47].

### Predictors of State Anxiety

The results of significant gender differences in state anxiety achieved particular significance in light of the regression analysis results indicating that gender indeed contributed to state anxiety prediction. Both results emphasize the importance of considering gender as a factor that plays a role in dealing with stressful events and relates to the mental health of older persons in challenging periods as the COVID-19 pandemic.

The finding in this study that daily functional self-actualization predicts 41% of state anxiety, and that specific aspects of functional cognition (eg, complex activities) predict another 3% is particularly interesting. Positive effects were previously reported between cognitive functioning and mild and moderate state anxiety among older people in routine times [12]. Knowledge of these relationships between anxiety and the common available resources of daily functional self-actualization and functional cognition related to gender differences, potentially obtainable in part through self-awareness and self-reflection, seems to play an important role in participation. Satisfactory participation in daily life activities promotes well-being and increases individuals’ sense of purpose and meaning [48]. The level of interest, value, and personal causation in everyday activities [46], especially leisure, social, and physical activities, has been associated with life satisfaction and psychological well-being among older adults [49-54]. For instance, Soderhamn et al [30] conducted interviews among 11 adults aged 67 years and older to grasp the meaning of self-care actualization (eg, activities that improve, maintain, or restore health). They found that self-care, as well as the use of self-care resources to care for others, was meaningful due to the sense of control over life and the self-reflection and realization it provides [30]. We surmise that the use of web-based self-report questionnaires (such as the DLQ and DailyFA used in this study) may be a resource to achieve such self-reflection. If so, the use of web-based self-report questionnaires may enhance self-awareness, psychological autonomy, life control, health, and well-being [20,55,56].

### Limitations and Future Research

The results of this study should be interpreted in the context of several limitations. First, the DailyFA questionnaire was developed to address the urgent need to measure daily functional self-actualization during the COVID-19 lockdown period; more research is required to further establish the validity and reliability of the scale. Second, it was not our aim to compare precrisis and postcrisis results. Because anxiety and functioning may be correlated in general (and not specifically in relation to the COVID-19 pandemic), further studies are required to compare features in routine precrisis periods to features during the crisis to determine whether these study results depend upon the crisis period or reflect a global phenomenon among older adults. Third, we did not explore the potential effects of self-reflection that may have arisen as participants completed the web-based self-report questionnaires. Future studies should explore the effects of such questionnaires on respondents’ well-being.

### Conclusions

Without doubt, late life is a challenging period associated with forfeiture and changes in physical, emotional, and social domains. Recognizing a person’s resiliency resources while focusing on functional abilities in conjunction with mental health may help older adults turn adverse events into opportunities and increase personal growth and life satisfaction [57]. Physical and mental health status is essential for people to perceive their personal strengths, while psychological resources may help them deal with physical and mental impairments and new circumstances, such as the COVID-19 crisis [44].

## Abbreviations

DailyFA: Daily Functional Actualization
DLQ: Daily Living Questionnaire
ICF: International Classification of Functioning, Disability, and Health
STPI: State-Trait Personality Inventory
WHO: World Health Organization
